# Deprivation amplification revisited; or, is it always true that poorer places have poorer access to resources for healthy diets and physical activity?

**DOI:** 10.1186/1479-5868-4-32

**Published:** 2007-08-07

**Authors:** Sally Macintyre

**Affiliations:** 1 MRC Social and Public Health Sciences Unit, Glasgow, G12 8RZ, UK

## Abstract

**Background:**

It has commonly been suggested (including by this author) that individual or household deprivation (for example, low income) is amplified by area level deprivation (for example, lack of affordable nutritious food or facilities for physical activity in the neighbourhood).

**Discussion:**

The idea of deprivation amplification has some intuitive attractiveness and helps divert attention away from purely individual determinants of diet and physical activity, and towards health promoting or health damaging features of the physical and social environment. Such environmental features may be modifiable, and environmental changes may help promote healthier behaviors. However, recent empirical examination of the distribution of facilities and resources shows that location does not always disadvantage poorer neighbourhoods. This suggests that we need: a) to ensure that theories and policies are based on up-to-date empirical evidence on the socio-economic distribution of neighbourhood resources, and b) to engage in further research on the relative importance of, and interactions between, individual and environmental factors in shaping behavior.

**Summary:**

In this debate paper I suggest that it may not always be true that poorer neighbourhoods are more likely to lack health promoting resources, and to be exposed to more health damaging resources. The spatial distribution of environmental resources by area socioeconomic status may vary between types of resource, countries, and time periods. It may also be that the presence or absence of resources is less important than their quality, their social meaning, or local perceptions of their accessibility and relevance.

## Background

Since the mid 1990s there has been considerable interest in the relative importance of individual and environmental characteristics in influencing health and health related behaviors. An extensive literature on area variations in health has reviewed the traditional distinction between compositional and contextual explanations (the former referring to the nature of the residents of an area, the latter to the nature of the area) [1-5]. Most empirical studies have concluded that who you are (e.g. age, gender, race, social class) is the main predictor of health and health related behavior, but that where you live also matters [2]. This has been shown for total and coronary heart disease (CHD) mortality [6,7], CHD prevalence and risk factors [8], morbidity [9], depression [10], and diet, physical activity, smoking and alcohol consumption [11-13]. It has also been found that individual and area characteristics may interact [14,15]; for example, in the West of Scotland 'bad diet' was related to area deprivation, but only among more affluent households [12].

The observation that health and health related behaviors tend to be poorer in more disadvantaged areas even after controlling for individual characteristics has been associated with a broader idea that environmental characteristics in poorer areas are generally more detrimental to health and healthy living. We have previously described this as 'deprivation amplification', a process, applying across the whole range of environmental influences on health, by which disadvantages arising from poorer quality environments (for example, lack of good public transport) amplify individual disadvantages (for example, lack of private transport) in ways which are detrimental to health [16,17]. In our work in the early 1990s on two socially contrasting study areas in Glasgow, Scotland, we observed this for transport, shops, primary health care, and a range of perceived environmental incivilities such as litter, uneven pavements and graffiti [16]. The concept of 'deprivation amplification' is similar to that of the 'inverse care law' first propounded in relation to health care provision (this suggests that the availability of good medical care tends to vary inversely with the need for it in the population served) [18].

The fact that levels of obesity, physical activity and poor diet are higher in more deprived neighbourhoods has also been associated with the development of the concept of obesogenic environments, that is, environments which promote excessive food intake and discourage physical activity [19-22]. A somewhat separate body of research on diet in the UK has focused less on obesity and excessive food intake and more on lack of access to nutritious, affordable, food such as fruit and vegetables in poorer neighbourhoods [23,24]. Both bodies of literature imply some sort of deprivation amplification, in that access (or lack of it) to environmental resources is seen to compound individual advantage or disadvantage.

## Discussion

### Theoretical and policy utility of the concept of deprivation amplification

The concept of deprivation amplification is important both for theorising about the influences of personal and environmental attributes (and interactions between them), and for policy. A research focus on individual characteristics would involve examining the role of socio-demographic variables such as age, sex, income, housing, and car access; and of personal variables such as taste preferences, cognitions, motivation, awareness, efficacy, socialisation and experiences. A policy focus on individual characteristics would involve trying to improve personal resources, whether material, via for example income supplementation, or attitudinal or motivational, for example as suggested in cognitive behavioral theories [25-27]. A policy focus on area characteristics, by contrast, might involve trying to improve the geographical distribution of and access to health promoting amenities and resources, via strategies such as urban planning and zoning regulations [20,28-30].

The geographical and epidemiological research cited above, suggesting a residual influence of area of residence after controlling for individual characteristics, provided a useful impetus both for empirical research into what features of areas might influence health and health related behaviors, and for thinking about policies to improve environments [1,17,31]. Research on nutrition and physical activity has increasingly suggested that areas in which poorer people live may have poorer environments and less access to health promoting amenities, for example:

*'Poorer neighbourhoods tend to have fewer recreation amenities, be less safe, and have a higher concentration of fast food outlets' *[21] p133.

*'There is long established evidence that the provision of health-related facilities is often inversely associated with population need*. [32] p128

*'This [review] paper provides evidence that economically disadvantaged and racial/ethnic minority populations have substantial environmental challenges to overcome to become physically active, to acquire healthy dietary habits, and maintain a healthy weight*.'[33] pS30

Policy recommendations in relation to reducing inequalities in health have also given increasing recognition to the possible effects of deprivation amplification, e.g. the Independent Enquiry into Inequalities in Health in the UK suggested that;

*'People on low incomes eat less healthily partly because of cost, rather than lack of concern or information. Therefore increased availability of affordable "healthy" food should lead to improved nutrition in the least well off... Town and country planning regulations could be amended or emphasised to ensure that development of retail food outlets do not have an adverse effect on those most vulnerable to poor nutrition.'*[24] pps 65–66

While for sociologists such as myself this emphasis on environmental barriers and promoters expresses a welcome antidote to the view that differences between areas are solely due to differences in the personal characteristics of the residents, it is necessary to note that such policies may be misguided if based on poor empirical information.

### An example: food deserts

The idea that poorer areas have worse environmental resources has become commonplace both in theory and policy in some fields. One example of this in the UK is the concept of food deserts. The term food deserts was coined in the mid-1990s to describe: 'those areas of inner cities where cheap, nutritious food is virtually unobtainable. Car-less residents, unable to reach out-of-town supermarkets, depend on the corner shop where prices are high, products are processed and fresh fruit and vegetables are poor or non-existent' [34] The concept has been widely disseminated and used by researchers and food activists, and incorporated into UK government policy on nutrition, inequalities in health (see above) and social exclusion [24,35,36]. However, even at the time at which this term was receiving wide currency, there was very little empirical evidence about the existence of food deserts. Three studies in the UK often cited as proving the existence of food deserts mainly concentrated on the price of healthy compared with unhealthy food, and did not examine the location of food outlets. This led Cummins and Macintyre to describe the concept of food deserts as being a 'factoid', an assertion that is repeated so often that it is believed to be true [37].

More extensive empirical investigations of food deserts in the UK have since found very little evidence that areas with large proportions of deprived residents are poorly served by retail food stores. In Glasgow we found that large multiple supermarkets were actually *more likely *to be located in deprived neighbourhoods, and that when there were differences in the pricing of foodstuffs, these tended to be slightly *cheaper *in poorer areas [38,39]. Similar findings have been reported in the UK [40] and elsewhere [41,42] Another study in the UK found that despite government beliefs that low income groups have difficulties in accessing and affording fruit and vegetables, few low income participants said that they experienced any difficulty visiting supermarkets, or perceived any problems in the choice of shops, or of fruit and vegetables, in their local area [43].

### Deprivation amplification revisited

In our work in Glasgow, we have other evidence that environmental resources are not always distributed in the way that might be suggested by the concept of deprivation amplification. In the original paper in which we suggested systematic differences in neighbourhood characteristics between two socially contrasting areas, we did note some exceptions or subtleties. While we showed that in total there were more official recreation facilities for physical activity in the more socially deprived locality, we also observed that there were three recreation centres and one sports centre in the poorer and none in the richer locality, and the same number of sports halls and swimming pools in each. There were nearly twice as many community health clinics, three times as many general practices, twice as many general practitioners, three times as many dentists, four times as many opticians, and one and a half times as many pharmacies in the richer compared to poorer locality, conforming to the deprivation amplification or inverse care law patterns. However a larger proportion of practices in the poorer locality operated out of purpose-built accommodation, more of the general practitioners there had qualified relatively recently, and more of its practices had attached community nurses and provided special clinics (all potential markers for better quality services). The frequency of street sweeping and de-littering was higher in the poorer than in the richer area; although there was a greater incidence of crimes against the person in the poorer area, there was a greater incidence of crimes against commercial premises and cars in the richer area; and residents of the richer area were more likely to complain about smells and fumes (emanating from sewage works and a rape seed processing factory) [16]. Thus not all local resources or experiences were located to the detriment of the more deprived locality.

Perhaps more relevant to this journal, it has recently been observed in Glasgow that the proportion of the population living within 300 meters of a green space greater than two hectares in size is greater among the more deprived. For example, in areas where less than a quarter of the population are classified as poor, 33.3 percent live within 300 meters of such a green space, compared with 52.2 percent in areas in which between a quarter and a half of the residents are poor, and 61.3 percent in areas where over half the residents are poor [44]. Thus those in poorer areas have better access to green spaces in which to walk, play, or take their children. We have recently found that in Glasgow the mean number of public outdoor playgrounds per thousand children is lower in more affluent than in more deprived areas [45]. Of course, neither access to public green spaces nor outdoor children's playgrounds may compensate for there being fewer or smaller private gardens in poorer areas; but nevertheless our earlier idea that poorer areas would have objectively worse provision does not seem to be borne out in the case of these two potentially physical activity promoting resources.

We have also examined fast food outlets (i.e. those providing counter-only service of energy dense foods) in Glasgow. We found these were not concentrated in poorer residential areas, but rather in the central business district, West End, retail parks, and along arterial roads. Restaurants and cafes followed broadly the same pattern [46]. This, combined with our earlier findings about food retail outlets, suggests that in Glasgow, poorer areas are not necessarily deficient in access to affordable food meeting current nutritional guidelines (for example, fresh fruit and vegetables which are usually cheaper and of better quality in supermarkets), nor are they particularly exposed to fast food outlets selling high-fat, high salt, energy dense food at low prices.

Our findings in Glasgow differ from some, and are similar to others, from elsewhere. For example, in relation to the location of fast food outlets our findings differ from those reported for Melbourne and New Orleans, which showed greater exposure to fast food outlets in poorer or black areas [47-49], and we have recently shown that the density of the 'big four' chain restaurants is greater in more deprived areas in England and Scotland [50,51].

In relation to supermarkets and grocery stores, in Brisbane there were minimal or no socio-economic differences in food shopping infrastructure [42], in Alameda County there was no correlation between neighbourhood level socio-economic status (SES) and commercial stores [52], and the Competition Commission found that since changes in planning regulations in the 1990s there was no evidence of lack of access to supermarkets in poorer areas in the UK [53]. In the South East of the Netherlands there was, as in Glasgow, increased proximity to food shops with increasing socio-economic disadvantage [41].

In contrast, in Mississippi, North Carolina, Maryland and Minnesota, there were more supermarkets, and gas stations with convenience stores, in wealthier neighbourhoods, and four times more supermarkets in white neighbourhoods compared to black neighbourhoods [54]; in metropolitan Detroit the nearest supermarket was significantly further away in neighbourhoods with a high proportion of African-Americans, and in the most impoverished neighbourhoods [55]; and in the Minneapolis and St Paul metropolitan area there were fewer large chain grocery stores in poor areas [56]. In Melbourne, Australia, more advantaged areas had closer access to supermarkets [49]. This suggests both between-country and over-time variations in the distribution of food outlets, and in particular that the USA may be unusual in the degree to which those in poorer neighborhoods and/or higher concentrations of African Americans have less access to a range of healthy foods and more exposure to energy dense fast foods [57].

Some findings relating to physical activity resources are similar to ours in Glasgow. Outdoor children's playgrounds were more likely to be located in poorer areas in Amsterdam [58], Edmonton [59] and Boston [60]. In Perth, Australia, poorer people had significantly better access to objectively measured facilities for physical recreation than better off people [61]; in Melbourne there were no differences in the number or total area of free access, restricted access or sporting/recreation open spaces by neighbourhood SES [62]; and similarly, the GLOBE study in the Netherlands found no significant differences by neighbourhood socioeconomic environment in proximity to sports facilities [41]. However, in England there was a significant negative association between neighbourhood deprivation and density of physical activity resources, with poorer places having poorer access [63], and in the USA low SES areas had significantly fewer free-for-use resources for physical activity than high SES areas (though there was no difference by area SES in access to pay-for-use facilities)[64].

The Alameda County study found that while higher rates per thousand population of common commercial stores (including grocery stores, supermarkets, laundries/dry cleaners and pharmacies) were associated with predicted higher mortality, there was no correlation between population levels of SES and prevalence of commercial stores [52]. And finally, a study in New Zealand of 16 types of community resources found that 15 of these were more accessible in more deprived neighbourhoods (the exception being beaches), which led the authors to conclude that:

'*These results challenge the widely held, but largely untested view that areas of high social disadvantage have poorer access to community resources. Poor locational access to community resources among deprived neighbourhoods in New Zealand does not appear to be an explanation for poorer health in these neighbourhoods'*[65] p348.

Thus there does not seem to be any consistent pattern in whether or not resources are located to the disadvantage of households in poorer communities.

### Environmental 'goods' and 'bads'

The Alameda County Study findings mentioned above raise the question not only of whether we should re-examine the assumption that environmental resources are more likely to be found in better off areas, but also of whether having such goods in the immediate neighbourhood is actually health promoting. There is a common, but often implicit, view in much of the literature on environmental influences on nutrition and physical activity that certain resources (such as parks and supermarkets) are self evidently health promoting while others (such as fast food or alcohol outlets, or proximity to waste and derelict land) are self evidently health damaging. For example, Burns and Inglis recently wrote: 'Access to a major supermarket was used as a proxy for access to a healthy diet and fast food outlet as proxy for access to unhealthy food' [49], and in our own work on the location of fast food outlets we suggested that access to fast food might fuel obesity levels in poor areas [50,51]. However, supermarkets provide access to a range of foods at relatively low prices, so access to a supermarket could facilitate the bulk buying of high energy nutrient poor foods and therefore be health damaging. Also, the same resource could be health promoting for some people but health damaging for others; for example, proximity to vacant or derelict land might facilitate ball games among youths or free play among children (and thus be seen as health promoting) but simultaneously seem threatening and a deterrent to women joggers or elderly walkers. Similarly the social meaning and symbolic significance of some resources may also vary; for example, it has recently been reported that features such as woodlands, which are often seen to be health promoting, can be see as 'scary' by some people [66]. Treating some resources as environmental 'goods' and others as environmental 'bads' may therefore be unduly naïve and simplistic.

### Evidence based theory and policy

It is important that theories and policies about nutrition and physical activity behavior are based on up-to-date, rigorous, empirical evidence. A key issue in both theory and policy in this field is the relationship between individual and environmental attributes [21,25,30], an issue which I do not have space here to review. I do not wish to suggest that there are no environmental disincentives to healthy nutrition and physical activity in more disadvantaged areas. However, I think we need to look carefully at some of our assumptions, including those which suggest that such environmental disincentives are commonly present and might be able to explain poorer nutritional and physical activity behaviors in more disadvantaged areas.

In revisiting the concept of deprivation amplification, I suggest that we need to consider broader socio-economic and cultural contexts, and also the history of urban and rural planning and the design of the built environment. Patterns of residential segregation may differ markedly between the USA and Australia, where cities are in general more sprawling, and in the UK and Western Europe, where they may be more dense. Residential segregation along racial lines [67], and the separation of different activities through zoning laws [68,69], may also be more marked in the USA than elsewhere.

It may be that in some societies or in some historical periods more disadvantaged areas have relatively poor access to nutritious food and opportunities for physical activity. For example, the peripheral public housing estates which were built to re-house slum inhabitants after the Second World War in Glasgow were lacking many basic community facilities including grocery stores, but changes in the economics of retailing and in planning regulations in the UK mean that the big supermarket chains are now increasingly locating in both inner-city and peripheral public housing areas [38,53]. Equally, our present patterns of provision may reflect philanthropic or public attempts in earlier periods to provide better facilities, such as playgrounds or community centres, for the poor [58,60]. Residents of rich suburbs or gated communities may not wish to have commercial or publicly available facilities in their vicinity, and their greater ability to resist attempts to site such facilities near them may mean they are more likely to be located in less affluent localities. Thus there are understandable contextual reasons for a variety of distributional patterns, and it would be sensible not to assume that environmental resources are more likely to be concentrated in better off areas and unavailable to those in poorer areas. The key point is that the location of such resources in relation to personal household disadvantage is an empirical question.

There are other important issues. Most of the literature cited above has counted and mapped resources such as playgrounds or fast food outlets, and expressed their location in relation to neighbourhoods defined by postal or electoral boundaries. While it may be true that in quantitative terms poorer neighbourhoods may not be disadvantaged by the location of specific resources, it may be that they are disadvantaged in terms of quality; for example outdoor playgrounds may be less attractive, have less equipment or have equipment in greater need of repair, or be seen as more dangerous, in poorer areas (we are exploring this possibility by auditing the quality of playgrounds in areas of different SES in Glasgow). It is also possible that people are influenced in their behaviors by the availability of resources in locations other than their immediate residential environment (for example, shopping in supermarkets, or using a park, near to their place of work or child's school).

Another issue is whether the location of facilities such as supermarkets in poorer areas is designed to meet, or actually meets, demand in the immediate area; such facilities may actually be patronised by customers from richer areas, and not be seen by locals as being appropriate to them. A study in Glasgow of the opening of a large chain supermarket in a poor neighbourhood that previously lacked one suggests that the main beneficiaries may have been people from outside the area who switched to that supermarket, rather than locals who continued to shop in smaller local shops and/or did not perceive the supermarket to be designed for them [70,71]. Similarly, fast food outlets might not be targeted at those who live in close proximity, but at those working or shopping locally, using local bars or cinemas, or passing by [46,72]. Finally, there is the important issue of whether it is the actual, objectively measured, presence or absence of facilities that is most likely to influence behavior, or the perceived or symbolic presence or absence of facilities.

## Conclusion

In conclusion, although we have used the term 'deprivation amplification' since the early 1990s to describe what we then considered to be a common pattern in which lack of community resources amplifies household poverty, I now believe that this pattern may be less standard than we initially suggested. Whether or not such a pattern is observed will depend on the resource in question, the regional or national context, and the historical epoch.

## Competing interests

The author(s) declare that they have no competing interests.
